# Pericapsular Nerve Group (PENG) Block versus Supra-Inguinal Fascia Iliaca Compartment Block for Total Hip Arthroplasty: A Randomized Clinical Trial

**DOI:** 10.3390/jpm12030408

**Published:** 2022-03-06

**Authors:** Yong Seon Choi, Kwan Kyu Park, Bora Lee, Won Seok Nam, Do-Hyeong Kim

**Affiliations:** 1 Department of Anesthesiology and Pain Medicine, Anesthesia and Pain Research Institute, Yonsei University College of Medicine, 50-1 Yonsei-ro, Seodaemun-gu, Seoul 03722, Korea; yschoi@yuhs.ac (Y.S.C.); dreamkaist@yuhs.ac (B.L.); galaxy995@yuhs.ac (W.S.N.); 2 Department of Orthopedic Surgery, Yonsei University College of Medicine, 50-1 Yonsei-ro, Seodaemun-gu, Seoul 03722, Korea; kkpark@yuhs.ac

**Keywords:** arthroplasty, hip surgery, nerve block, postoperative analgesia

## Abstract

This study compared the effects of the pericapsular nerve group (PENG) block and supra-inguinal fascia iliaca compartment block (FICB) on postoperative analgesia and quadriceps strength following total hip arthroplasty under general anesthesia. A total of 58 patients were randomized to receive either PENG block (PENG group) or supra-inguinal FICB (FICB group) following anesthetic induction. The primary outcomes were the postoperative pain scores. Patients were randomized to receive either PENG block or supra-inguinal FICB following anesthetic induction. Pain scores at rest and with movement were assessed preoperatively, at the postanesthesia care unit (only at rest), and at 6, 24, 36, and 48 h postoperatively. Opioid consumption was also assessed for 48 h postoperatively. Quadriceps strength measurements were performed preoperatively, at 6, 24, and 36 h postoperatively. In total, 54 patients completed the study: 27 in the PENG group and 27 in the FICB group. Despite lower pain scores at rest in the PENG group at postoperative 6 and 24 h, there were no significant differences in the pain scores at rest and during movement between the two groups during postoperative 48 h in the linear mixed model analysis (*p* = 0.079 and *p* = 0.323, respectively). Cumulative opioid consumption up to postoperative 48 h was also similar in the two groups (*p* = 0.265). The changes in quadriceps strength measurements in the operative leg and the nonoperative leg were not significantly different between the groups (*p* = 0.513 and *p* = 0.523, respectively). The PENG block may have similar analgesic efficacy to the supra-inguinal FICB. No difference was detected in the quadriceps strength between the patients receiving these two blocks.

## 1. Introduction

Adequate pain management following total hip arthroplasty (THA) is crucial for early ambulation and patient satisfaction [1,2]. However, owing to the complexity of the innervation of the hip joint, the optimal regional analgesia technique for THA remains controversial [3].

A supra-inguinal fascia iliaca compartment block (FICB), a new approach to FICB, provides better spread under the fascia iliaca while deposing local anesthetic more cranially, compared with the infra-inguinal approach [4,5,6,7]. Desmet et al. demonstrated that the supra-inguinal FICB resulted in reduced morphine consumption and pain scores following THA [8]. However, despite these promising results, obturator nerve block has not been clinically proven [7]. Moreover, the supra-inguinal FICB possesses a potential risk of quadriceps weakness, which could hamper early ambulation.

The anterior hip capsule is innervated by the femoral, accessory obturator, and obturator nerves [9]. According to recent anatomical studies, iliopubic eminence and inferomedial acetabulum were suggested as relevant bony landmarks to block the articular branches from these three nerves [10]. Moreover, a greater role of the femoral nerve and the accessory obturator nerve in the anterior hip innervation has also been noted [10]. These anatomical findings led Girón-Arango et al. to introduce a new technique for selective blockade of the articular branches from the femoral, accessory obturator, and obturator nerves [11]. This pericapsular nerve group (PENG) block has demonstrated sufficient analgesic effect, with reduced pain scores and no quadriceps weakness in patients with hip fracture [11]. However, in patients undergoing THA under general anesthesia, studies comparing the PENG block and the supra-inguinal FICB, two recently introduced regional analgesia techniques, are lacking.

In this randomized clinical study, we tested the hypothesis that the PENG block would provide better analgesia and result in less quadriceps muscle weakness when compared with the supra-inguinal FICB following THA. Our primary outcomes were the postoperative pain scores, and the secondary outcome measures included opioid consumption and degree of quadriceps weakness.

## 2. Materials and Methods

### 2.1. Study Population

This prospective, randomized clinical study was performed at the Severance Hospital, Yonsei University Health System, Seoul, Korea, in accordance with the Declaration of Helsinki. The study protocol was approved by the Institutional Review Board and Hospital Research Ethics Committee of Severance Hospital, Yonsei University Health System (#4-2020-0417), on 4 June 2020, and registered at ClinicalTrials.gov (NCT04426045) on 11 June 2020. Written informed consent was obtained from all the participants of the trial. Patients aged 19 years or older with an American Society of Anesthesiologists physical status I–III, who were scheduled for elective, unilateral THA under general anesthesia, were enrolled in this study between July 2020 and June 2021. Exclusion criteria were allergy or intolerance to any of the drugs used in the study, liver failure, renal insufficiency (estimated glomerular filtration rate < 15 mL/min/1.73 m^2^), known or suspected coagulopathy, pre-existing neurologic or anatomic deficits in the lower extremities, cognitive impairment with difficulties in pain evaluation, and severe psychiatric illness.

### 2.2. Study Design

Enrolled patients were randomly assigned to receive either the PENG block (PENG group) or the supra-inguinal FICB (FICB group) on the day of the surgery. An investigator who did not participate in either patient care or perioperative assessment carried out the group assignment according to a computer-generated randomization sequence, using a block randomization technique with a block size of 4 and a 1:1 ratio. Allocation results were concealed in sealed opaque envelopes, which were given to the anesthesiologist performing the PENG block and the FICB; this anesthesiologist was not involved with the study; therefore, surgeons, investigators, nursing staff, and patients were blinded to the group assignment during the study period.

### 2.3. Interventions

**Figure 1:**
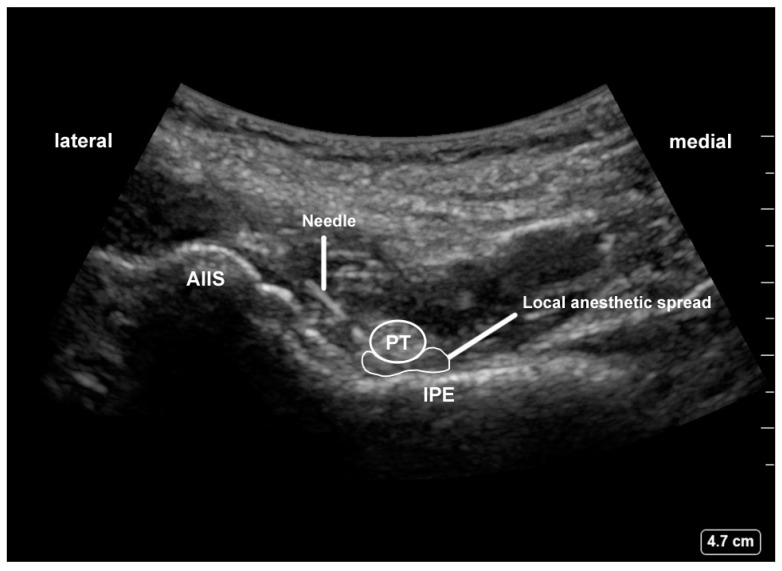
Pericapsular nerve group (PENG) block: AIIS, anterior inferior iliac spine; IPE, iliopubic eminence; PT, psoas tendon.

Nerve blocks were performed following anesthesia induction and before initiation of surgery. All blocks were performed by an experienced anesthesiologist otherwise not involved in the study. In the PENG group, the curvilinear low-frequency ultrasound probe (2–5 MHz; C60xp; SonoSite X-Porte; SonoSite Inc., Bothell, WA, USA) was placed over the line parallel to the inguinal ligament. It was subsequently rotated 45° to identify the anterior inferior iliac spine, the iliopubic eminence, and the psoas tendon. A 22-gauge, 80 mm echogenic needle was inserted in an in-plane approach to place the tip in the musculofascial plane between the pubic ramus posteriorly and the psoas tendon anteriorly, using the hydrodissection technique. Following negative aspiration, a total volume of 20 mL of ropivacaine 0.2% with epinephrine 1:200,000 was injected (Figure 1) [11].

In the FICB group, the linear 6–13 MHz ultrasound probe (HFL38xp; SonoSite Inc.) was placed over the inguinal ligament in the sagittal plane, inferior medially to the anterior superior iliac spine. Upon identifying the “bow-tie sign” formed by the sartorius and the internal oblique muscle by sliding medially and rotating the probe, a 22-gauge, 80 mm echogenic needle was introduced 1 cm cephalad to the inguinal ligament to place the needle tip in the space between the internal oblique and iliacus muscles, using the hydrodissection technique [8]. A total volume of 30 mL of ropivacaine 0.2% with epinephrine 1:200,000 was injected following negative aspiration.

### 2.4. Perioperative Management

All the patients received standardized general anesthetic management as practiced commonly in our hospital. Intravenous (IV) tranexamic acid 1000 mg and cefazolin 1 g were administered intraoperatively. In this study, a single surgical team performed all the THAs via a posterior approach. There was no surgeon-delivered periarticular infiltration during the surgery. At 30 min before the end of the surgery, IV fentanyl 1 μg/kg and palonosetron 0.075 mg were administered to the patient for postoperative analgesia and antiemetic effects, respectively. IV patient-controlled analgesia (PCA) was administered for 48 h postoperatively, which comprised fentanyl 7 μg/kg and palonosetron 0.075 mg (total volume including saline: 100 mL), delivered as 2 mL/h background infusion and 0.5 mL doses at upon the patient’s demand with 15 min of lockout time [12]. After the end of the surgery, the patients were transferred to the postanesthesia care unit (PACU). In the PACU, rescue analgesics (IV fentanyl 0.5–1.0 μg/kg) were administered when the pain score at rest was ≥4 or on patient request. In the ward, all patients received celecoxib 200 mg orally and acetaminophen 1 g intravenously every 12 h afterward. However, if the patients reported a persistent NRS pain score ≥4 or on patient request, rescue IV tramadol 25 mg was provided. In case of severe nausea or vomiting occurs, the patients were treated with 10 mg of metoclopramide. Patients received thromboembolism prophylaxis daily, with a direct factor Xa inhibitor for 4 weeks postoperatively. Patients were instructed to perform quadriceps exercise on the day of surgery and encouraged early ambulation following surgery.

### 2.5. Outcome Assessments

The primary outcome measures were pain scores at rest and during 45° passive flexion of the hip up to 48 h following surgery. We recorded the intensity of pain at rest and during 45° passive flexion of the hip with the ipsilateral knee flexed 45° preoperatively, at PACU (only at rest), and at 6, 24, 36, and 48 h postoperatively using an 11-point numeric rating scale (NRS: 0 = no pain, 10 = worst imaginable pain). The secondary outcomes included opioid consumption and quadriceps muscle strength. Opioid consumption during 0–6, 6–24, and 24–48 h postoperatively and cumulative opioid consumption at 6, 24, and 48 h following surgery were recorded. The consumption of the different types of postoperative opioids including IV PCA-administered fentanyl was converted to oral morphine equivalents [13]. The Quadriceps strength of each patient was tested using a handheld dynamometer, a reliable and valid instrument assessing muscle strength [14]. By placing patients in a supine position with a cushion underneath their knee, 45° passive flexion of the hip and ipsilateral knee was maintained, and the dynamometer was placed on the anterior side of the ankle between the malleoli. Patients were instructed to extend their legs two times each, with a 30 s pause between each attempt. The measurement was made preoperatively, at 6, 24, and 36 h postoperatively. The following perioperative data were also collected: the total amount of fentanyl and remifentanil used during anesthesia, operation and anesthesia time, length of PACU stay, time to first standing and ambulation, patient satisfaction score (using a scale of 0–10, 10 being the most satisfied) at 48 h following surgery, length of hospital stay, and adverse events such as local anesthetic toxicity, falls, nausea, urinary retention, and dizziness. All outcomes and perioperative data were collected by an investigator blinded to the group allocation.

### 2.6. Statistical Analysis

No previous study has compared the pain scores between the patients receiving PENG block and supra-inguinal FICB for THA under general anesthesia. Referring to the sample size calculation of a previous study comparing the analgesic efficacy of supra-inguinal FICB and periarticular infiltration in patients of THA under general anesthesia, the standard deviation for the primary end point was assumed to be 2.5 [15]. A mean difference of 2.0 in pain scores between the groups was considered clinically significant [16,17]. To obtain a power of 0.80 (1-β) with an α of 0.05, the calculated sample size was 26 patients per group. To permit a dropout rate of 10%, the target sample size was 29 patients per group.

The normality of the data distribution was assessed using the Shapiro–Wilk test. Continuous variables were analyzed using the independent *t*-test or Mann–Whitney U test. Categorical variables were analyzed by the χ2 test or Fisher’s exact test. Values are presented as mean ± standard deviation, median (interquartile range), or the number of patients (proportion). The balance on patient and operation characteristics between the randomized groups was analyzed by calculating the standardized difference, defined as the difference in proportions or means divided by the pooled standard deviation. Serially measured variables were assessed using a linear mixed model with the patient indicator as a random effect, and group, time, and group-by-time interaction as fixed effects, adjusting for variables of patient and operation characteristics (sex, age, body mass index, ASA physical status, diabetes mellitus, preoperative nonsteroidal anti-inflammatory drugs use, chronic opiate use, diagnosis, surgical side, and operation time). An unstructured covariance structure was used. Bonferroni correction was applied to adjust for multiple comparisons. All analyses were performed using R version 4.0.3 (The R Foundation for Statistical Computing, Vienna, Austria), MedCalc version 20 (MedCalc, Ostend, Belgium), and SAS software version 9.4 (SAS Institute Inc., Cary, NC, USA). *p* < 0.05 was considered statistically significant.

## 3. Results

### 3.1. Patient and Operation Characteristics

**Figure 2:**
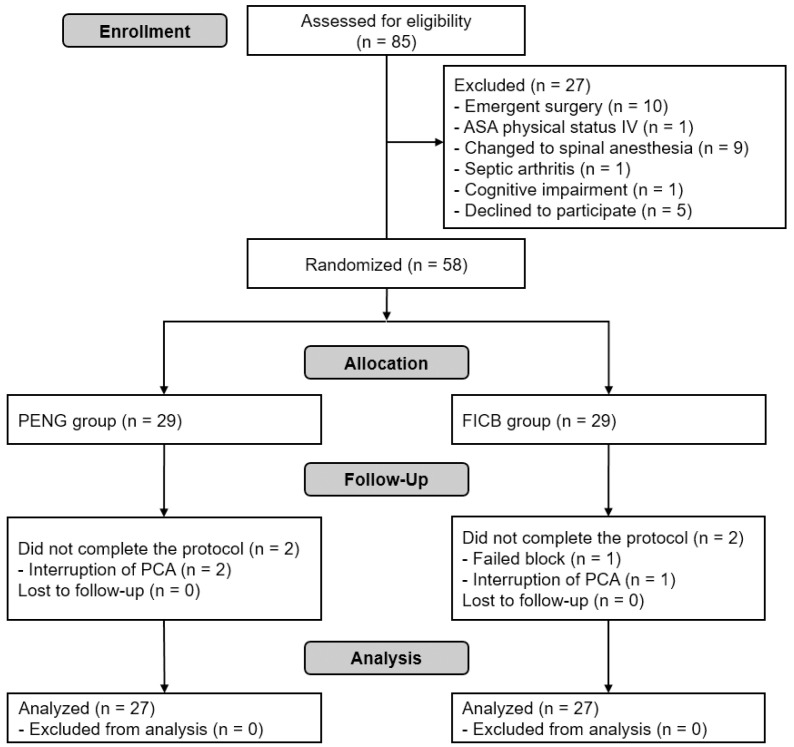
Flow diagram of patient selection: ASA, American Society of Anesthesiologists; FICB, supra-inguinal fascia iliaca compartment block; PCA, patient-controlled analgesia; PENG, pericapsular nerve group block.

Of the 85 patients assessed for eligibility, 27 were excluded. Thus, 58 patients were enrolled. Four were excluded from analysis due to interruption of PCA (*n* = 3; 2 in PENG group, 1 in FICB group) and failed block (*n* = 1, FICB group). Consequently, 54 patients were included in the final analysis (Figure 2). Patient and operation characteristics are detailed in Table 1.

### 3.2. Pain Outcomes

**Figure 3:**
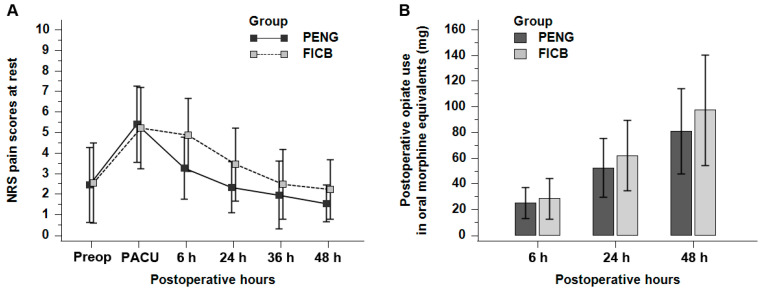
Pain intensity and opioid consumption: (**A**) NRS pain scores at rest over time. Data are expressed as mean ± standard deviation; (**B**) total opioid use over time. The bar chart displays the mean cumulative opioid consumption with standard deviation (error bars) at 6, 24, and 48 h following surgery. FICB, supra-inguinal fascia iliaca compartment block; NRS, numeric rating scale; PACU, postanesthesia care unit; PENG, pericapsular nerve group block.

Postoperative pain scores and opioid consumption are shown in Table 2 and Figure 3. There was no significant group-by-time interaction for the comparison of the NRS pain scores at rest up to postoperative 48 h between the PENG group and the FICB group (*p* = 0.079). NRS pain scores during movement were also not significantly different between the two groups when all time points were combined (*p* = 0.323). When a post hoc analysis was performed, the NRS pain scores at rest were lower in the PENG group than those in the FICB group at postoperative 6 and 24 h. Opioid consumptions during 0–6, 6–24, and 24–48 h following surgery were similar in the two groups (*p* = 0.728). Cumulative opioid consumption over time was also not significantly different between the groups (*p* = 0.265).

### 3.3. Quadriceps Strength

The dynamometer readings are shown in Table 3. The changes in quadriceps strength measurements in the operative leg over time, as well as the nonoperative leg, were not significantly different between the groups (*p* = 0.513 and *p* = 0.523, respectively). Quadriceps strength measurements in the operated leg decreased from preoperative values in both the PENG and FICB groups at each time point.

### 3.4. Intraoperative Anesthesia-Relevant Data and Postoperative Hospital Course

Table 4 summarizes intraoperative anesthesia-relevant data (total fentanyl and remifentanil dose, blood loss, and anesthesia time) and postoperative hospital course facts (nausea, and urinary retention, time to ambulation, length of hospital stay, and patient satisfaction). There were no block-related complications, such as local anesthetic toxicity, bleeding, or infection.

## 4. Discussion

In this randomized study, we did not find any significant differences in postoperative pain scores and opioid consumption up to postoperative 48 h between patients undergoing THA who received the PENG block and the supra-inguinal FICB. Moreover, we found that quadriceps strength in the operative leg decreased in both the PENG and FICB groups, and there were no significant differences between the groups.

The PENG block was introduced to alleviate pain by blocking only the articular branches innervating the anterior hip capsule, not the three main nerves of the lumbar plexus themselves, which results in sparing the motor function [11]. Lin et al. reported that patients undergoing hip fracture surgery who received PENG block experienced less postoperative pain than those who received femoral nerve block [18]. In patients undergoing THA, Pascarella et al. demonstrated that the PENG block reduced maximum pain scores and opioid consumption, compared with the no block group during postoperative 48 h [19]. However, Aliste et al. reported that between patients receiving the PENG block and those receiving the supra-inguinal FICB, no differences were found in static and dynamic pain scores during postoperative 48 h, as well as cumulative opioid consumption at 24 and 48 h after THA under spinal anesthesia [20]. Consistent with this previous report, the PENG block had comparable efficacy in terms of postoperative analgesia and opioid consumption when compared with the supra-inguinal FICB in patients undergoing THA under general anesthesia in this study.

According to a histologic study, the nociceptive fiber concentration is high in the anterior and superolateral regions of the hip joint capsule [21]. Therefore, regional analgesia techniques for hip surgery have mainly targeted the femoral and obturator nerve innervating the anterior hip capsule. A previous dye injection study suggested that 10 to 20 mL of injectate for the PENG block covered the articular branches of the obturator nerve [22]. However, these articular branches may not have been blocked sufficiently because they are close to the inferomedial acetabulum, away from the needlepoint. Likewise, although the supra-inguinal FICB spreads the injectate to the usual anatomical location of the obturator nerve [7], it is not yet clear whether the clinical block of the obturator nerve is achieved by this technique. Therefore, the main mechanism underlying the analgesic effect of the PENG block and the supra-inguinal FICB seems to be the blockage of the femoral nerve and its articular branches, which may explain why there were no differences in postoperative pain outcomes between patients who received the two blocks in this study. However, further anatomical and clinical studies are required on this issue.

Although there were no significant differences in the NRS pain scores through postoperative 48 h between the two groups when analyzed with a linear mixed model, the pain scores at 6 and 24 h following surgery were lower in the PENG group than those in the FICB group. Panzenbeck et al. demonstrated that pain at rest peaked up to 2 h following THA in patients receiving general anesthesia, and pain on movement was higher at 2 and 4 h following surgery, compared with spinal anesthesia [23]. Therefore, our results warrant studies to determine an optimal single-shot nerve block that aims to provide effective analgesia for patients of THA under general anesthesia in the early postoperative period (e.g., within 6 h after surgery).

Contrary to prior studies, our study did not find a significant difference in the quadriceps strength between the two groups. Aliste et al. recently reported that PENG block resulted in a lower incidence of quadriceps motor block at 3 and 6 h following THA, compared with the supra-inguinal FICB [20]. However, the quadriceps strength was not quantitatively measured using a dynamometer. Although superficial or medial local anesthetic injection of the target anatomical location while performing the PENG block was considered to lead to unexpected motor blockade [24,25], there was no serious difficulty in needle placement using PENG block in this study. Aliste et al. performed supra-inguinal FICB with 40 mL levobupivacaine 0.25% [20]. The studies by Desmet et al. and Gasanova et al. reporting the higher incidence of quadriceps motor block following supra-inguinal FICB used 40 mL of ropivacaine 0.5% and 60 mL of ropivacaine 0.5% as the injectate, respectively [8,15]. Although a direct comparison is impossible, our supra-inguinal FICB with 30 mL ropivacaine 0.2% possibly led to less quadriceps muscle weakness, which was speculated as one of the causes for the discrepancy between our study and that by Aliste et al. Moreover, motor function can be inhibited by postoperative pain and surgical factors, such as transient traction injury or tissue disruption. Therefore, pain and surgical insult as confounding factors could reduce the differences between the groups. Further, in-depth studies considering both pain and surgical factors are needed to determine the effect of PENG block on motor function and its recovery.

Recently presented recommendations for multimodal analgesia in THA included regional analgesic techniques such as single-shot FICB [26]. These regional techniques were recommended especially if patients had contraindications to basic analgesics or in patients with high expected postoperative pain. However, more lines of evidence are needed regarding regional techniques that provide adequate analgesia with early postoperative mobility, optimal functional recovery, and decreased postoperative morbidity under multimodal analgesic regimen [27]. The results of our study could aid in establishing multimodal analgesic protocols for THA patients.

In this study, no patient suffered from postoperative infection. Using a practical and efficient low-level disinfection technique and sterile barrier, the block-related infection rate following ultrasound-guided single-injection peripheral nerve block is extremely low [28]. However, the risk of postoperative infection could be high after the PENG block because the injection site overlaps the surgical field. Surgeons may express concern when they notice that anterior retractors are positioned, and a total hip prosthesis is implanted in the area where the block has been performed. An alternative to the PENG block following THA is intraoperative local infiltration analgesia in the surgical field under sterile conditions. This has been reported to be simple and effective [29], but further studies are needed to support its analgesic efficacy.

This study has certain limitations. First, one of the limitations of our study is the small study population. Although this study was a randomized study, there were differences in patient and operation characteristics between the two groups. This may have resulted due to the small number of patients. Therefore, serially measured outcomes were assessed using a linear mixed model adjusting for the variables of the patient and operation characteristics. However, further study with a large population is warranted. Second, the attending anesthesiologists were not blinded to the group assignment. However, the same and standardized general anesthetic protocol was implemented in all patients, and total intraoperative fentanyl and remifentanil doses were comparable between the two groups, respectively. Third, the difference in the volume and dose of the injectate between the two blocks could be a main confounding factor in our results. However, the goal of this study was to compare “PENG block” and “supra-inguinal FICB” rather than compare two different injection sites of local anesthetic. Fourth, a sensory assessment was not conducted. Since blocks were performed following anesthesia induction, the sensory exam could not be conducted preoperatively. The sensory block assessment may be difficult in the immediate postoperative period, owing to the confounding effects of postoperative pain and residual opioids, as well as the effects of general anesthesia. Moreover, previous studies showed that the success of FICB was not determined through the confirmation of the blockade of nerves of the lumbar plexus [4,8,15], and the sensory exam of the articular branches of these nerves is impossible following PENG block. Both blocks, as field block and plane block, were identified as completed by confirming the proper needle position and subsequent appropriate spread of injectate on real-time ultrasound view. The importance of sensory assessment after PENG block is clinically identifying the unintended spread of local anesthetic. Further studies are warranted to define the success of PENG block and FICB and elucidate the optimal dose, volume, and concentration of local anesthetic.

## 5. Conclusions

In conclusion, in the patients undergoing THA under general anesthesia, although the pain scores at rest were lower in the PENG group than those in the FICB group at postoperative 6 and 24 h, respectively, there were no significant differences in the pain scores between the patients receiving the PENG block and the supra-inguinal FICB throughout 48 h postoperatively. No differences in the opioid consumption and the quadriceps strength were also detected between the PENG and FICB groups. Additional studies are required to clarify the benefits of postoperative clinical pathways afforded by these two blocks in THA patients.

## Figures and Tables

**Table 1:** Patient and operation characteristics.

	PENG (*n* = 27)	FICB (*n* = 27)	ASD
Female	13 (48.1)	11 (40.7)	0.150
Age (years)	61.0 (48.5–72.0)	63.0 (52.0–71.0)	0.179
BMI (kg/m^2^)	25.8 ± 3.0	25.0 ± 3.9	0.244
ASA physical status			
I	5 (18.5)	2 (7.4)	0.335
II	15 (55.6)	22 (81.5)	0.581
III	7 (25.9)	3 (11.1)	0.389
Diabetes mellitus	7 (25.9)	2 (7.4)	0.513
Preoperative NSAIDs use	15 (55.6)	12 (44.4)	0.224
Chronic opiate use	11 (40.7)	7 (25.9)	0.318
Diagnosis			
Osteoarthritis	9 (33.3)	10 (37.0)	0.078
Avascular osteonecrosis	17 (63.0)	15 (55.6)	0.151
Implant loosening	1 (3.7)	2 (7.4)	0.162
Surgical side (right)	12 (44.4)	7 (25.9)	0.395
Operation time (min)	69.0 (57.0–78.0)	71.0 (60.0–80.5)	0.318

Data are presented as mean ± standard deviation, median (interquartile range), or number of patients (%). ASA, American Society of Anesthesiologists; ASD, absolute standardized difference; BMI, body mass index; FICB, supra-inguinal fascia iliaca compartment block; NSAIDs, nonsteroidal anti-inflammatory drugs; PENG, pericapsular nerve group block.

**Table 2:** Postoperative pain scores and opioid consumption.

	PENG (*n* = 27)	FICB (*n* = 27)	Difference (95% CI)	*p*_Group×Time_ *	Adjusted *p* †
NRS pain scores at rest				0.079	
Preoperative	2.6 (0.4)	2.8 (0.5)	−0.2 (−1.2 to 0.8)		>0.999
At PACU	5.6 (0.4)	5.5 (0.5)	0.1 (−1.0 to 1.2)		>0.999
6 h after surgery	3.4 (0.3)	5.1 (0.5)	−1.7 (−2.7 to −0.7)		0.004
24 h after surgery	2.5 (0.3)	3.7 (0.5)	−1.2 (−2.0 to −0.4)		0.022
36 h after surgery	2.1 (0.3)	2.7 (0.4)	−0.6 (−1.5 to 0.3)		>0.999
48 h after surgery	1.7 (0.3)	2.5 (0.4)	−0.7 (−1.5 to 0.0)		0.298
NRS pain scores during movement ‡				0.323	
Preoperative	5.7 (0.3)	6.0 (0.5)	−0.4 (−1.3 to 0.6)		>0.999
6 h after surgery	5.8 (0.3)	7.0 (0.5)	−1.2 (−2.2 to −0.1)		0.159
24 h after surgery	5.0 (0.3)	6.0 (0.5)	−1.1 (−2.0 to −0.2)		0.116
36 h after surgery	5.0 (0.3)	5.1 (0.6)	−0.3 (−1.4 to 0.7)		>0.999
48 h after surgery	4.3 (0.4)	4.5 (0.5)	−0.2 (−1.1 to 0.7)		>0.999
Opioid consumption (mg) §				0.728	
0–6 h after surgery	29.0 (2.9)	34.2 (5.1)	−5.2 (−13.3 to 2.8)		0.615
6–24 h after surgery	31.1 (2.6)	39.1 (5.1)	−8.0 (−16.4 to 0.5)		0.195
24–48 h after surgery	32.4 (2.9)	41.0 (5.4)	−8.5 (−18.7 to 1.8)		0.313
Cumulative opioid consumption (mg) §				0.265	
6 h after surgery	32.1 (5.6)	38.3 (9.2)	−6.2 (−17.6 to 5.2)		0.864
24 h after surgery	59.5 (5.0)	71.8 (9.5)	−12.3 (−26.7 to 2.1)		0.282
48 h after surgery	88.1 (5.0)	107.1 (10.6)	−19.0 (−38.8 to 0.8)		0.180

Data are presented as estimated mean (standard error): CI, confidence interval; FICB, supra-inguinal fascia iliaca compartment block; NRS, an 11-point numeric rating scale (0 = no pain, 10 = worst imaginable pain); PACU, postanesthesia care unit; PENG, pericapsular nerve group block. * *p* value of the group-by-time interaction in the linear mixed model. † *p* value was adjusted using the Bonferroni correction for multiple comparisons. ‡ Pain scores during 45° passive flexion of the hip with the ipsilateral knee flexed 45°. § Opioid consumption was converted to mg of oral morphine equivalents.

**Table 3:** Quadriceps muscle strength measurements.

	PENG (*n* = 27)	FICB (*n* = 27)	Difference (95% CI)	*p*_Group×Time_ *	Adjusted *p* †
Operative leg (kgf)				0.513	
Preoperative	10.6 (0.7)	11.3 (1.4)	−0.7 (−2.9 to 1.4)		>0.999
6 h after surgery	5.7 (1.0)	4.9 (1.3)	0.9 (−1.8 to 3.5)		>0.999
24 h after surgery	7.0 (0.7)	7.3 (1.3)	−0.2 (−2.1 to 1.6)		>0.999
36 h after surgery	7.7 (0.8)	7.1 (1.2)	0.6 (−1.4 to 2.6)		>0.999
Nonoperative leg (kgf)				0.523	
Preoperative	12.2 (1.0)	12.9 (1.6)	−0.8 (−2.9 to 1.4)		>0.999
6 h after surgery	9.9 (1.1)	11.8 (1.5)	−2.0 (−4.8 to 0.9)		0.709
24 h after surgery	10.5 (1.2)	12.1 (1.6)	−1.5 (−4.2 to 1.1)		>0.999
36 h after surgery	10.7 (1.1)	12.0 (1.6)	−1.4 (−4.1 to 1.3)		>0.999

Data are presented as estimated mean (standard error): FICB, supra-inguinal fascia iliaca compartment block; kgf, kilogram-force unit; PENG, pericapsular nerve group block. * *p* value of the group-by-time interaction in the linear mixed model. † *p* value was adjusted using the Bonferroni correction for multiple comparisons.

**Table 4:** Intraoperative and postoperative data.

	PENG (*n* = 27)	FICB (*n* = 27)	*p*
Intraoperative			
Total dose of fentanyl (μg/kg)	0.8 ± 0.1	0.7 ± 0.2	0.857
Total dose of remifentanil (μg/kg)	5.4 (4.4–6.3)	5.2 (4.4–7.0)	0.580
Blood loss (mL)	100.0 (100.0–200.0)	100.0 (100.0–200.0)	0.858
Anesthesia time (min)	105.0 (90.0–130.0)	110.0 (97.5–125.0)	0.340
Postoperative			
PACU stay (min)	40.0 (30.0–48.0)	44.0 (31.5–52.0)	0.625
Nausea	7 (25.9)	5 (18.5)	0.743
Urinary retention	2 (7.4)	1 (3.7)	0.999
Time to ambulation (h)	21.1 (17.8–24.1)	23.5 (20.2–29.0)	0.085
Length of hospital stay (day)	4.0 (4.0–4.0)	4.0 (4.0–4.0)	0.475
Patient satisfaction score	8.0 (6.0–8.0)	7.0 (5.0–8.0)	0.509

Data are presented as mean ± standard deviation, median (interquartile range), or number of patients (%): FICB, supra-inguinal fascia iliaca compartment block; PACU, postanesthesia care unit; PENG, pericapsular nerve group block.

## Data Availability

The data presented in this study are available from the corresponding author upon reasonable request.
